# Dynamic oxygen assessment techniques enable determination of anesthesia’s impact on tissue

**DOI:** 10.21203/rs.3.rs-4751349/v1

**Published:** 2024-08-27

**Authors:** Megan A. Clark, Armin D. Tavakkoli, Arthur F. Petusseau, Augustino V. Scorzo, Alireza Kheirollah, Scott C. Davis, Rendall R. Strawbridge, Petr Bruza, Brian W. Pogue, David J. Gladstone, P. Jack Hoopes

**Affiliations:** Dartmouth College; Dartmouth College; Dartmouth College; Dartmouth College; Dartmouth College; Dartmouth College; Dartmouth College; Dartmouth College; University of Wisconsin–Madison; Dartmouth College; Dartmouth College

## Abstract

Tissue oxygenation is well understood to impact radiosensitivity, with reports demonstrating a significant effect of breathing condition and anesthesia type on tissue oxygenation levels and radiobiological response. However, the temporal kinetics of intracellular and extracellular oxygenation have never been quantified, on the timescale that may affect radiotherapy studies. C57BL/6 mice were anesthetized using isoflurane at various percentages or ketamine/xylazine (ket/xyl: 100/10 mg/kg) (N = 48). Skin pO_2_ was measured using Oxyphor PdG4 and tracked after anesthetization began. Oxyphor data was validated with relative measurements of intracellular oxygen via protoporphyrin IX (PpIX) delayed fluorescence (DF) imaging. Ex vivo localization of both PdG4 Oxyphor and PpIX were quantified. Under all isoflurane anesthesia conditions, leg skin pO2 levels significantly increased from 12–15 mmHg at the start of anesthesia induction (4–6 minutes) to 24–27 mmHg after 10 minutes (p < 0.05). Ketamine/xylazine anesthesia led to skin pO2 maintained at 15–16 mmHg throughout the 10-minute study period (p < 0.01). An increase of pO2 in mice breathing isoflurane was demonstrated with Oxyphor and PpIX DF, indicating similar intracellular and extracellular oxygenation. These findings demonstrate the importance of routine anesthesia administration, where consistency in the timing between induction and irradiation may be crucial to minimizing variability in radiation response.

## 1. Introduction

In conventional radiation therapy, it is well understood that molecular oxygen acts as a radiosensitizer, as described by the oxygen fixation hypothesis and oxygen enhancement ratio (OER)[1]. This tissue radiosensitivity is dependent on relative oxygenation of that tissue, with the highest dependance between 0 mmHg and 10 mmHg, plateauing around 20 mmHg[2]. As *in vivo* studies have been the backbone of radiation therapy (RT) research motivating clinical translation, standardization of practices impacting radiosensitivity are crucial to identify the underlying mechanism under investigation. With tissue oxygenation dramatically impacting RT results, it points to an important parameter to better understand.

Reports of the effect of tissue oxygenation are important because a vast majority of animal irradiation experiments are conducted under anesthesia, and anesthetic practices vary widely between research groups. Where volatile inhaled anesthetics such as isoflurane or sevoflurane are used, choice of carrier gas, as well as concentration, flow rate, and duration of induction and maintenance can have a meaningful difference on oxygenation levels. Even when consistent induction and maintenance protocols are used, particularly for rodents, depth of anesthesia is rarely monitored owing to the time, cost, and effort required to intubate or obtain telemetry in small animals[3].

Injectable anesthetics such as ketamine/xylazine (ket/xyl) or barbiturate cocktails allow for a more tailored approach to anesthesia via weight-based dosing. Still, cocktail composition and dosing practices vary between researchers[4]. Moreover, different classes of injectable anesthetics such as dissociative/sedative-hypnotic (e.g. ketamine, propofol), opioid (e.g. buprenorphine), and benzodiazepine/barbiturates have vastly different pharmacokinetics and pharmacodynamics, and therefore varying effects on physiological parameters such as tissue oxygenation.

Prior studies have sought to characterize the oxygenation status of rodents under anesthesia. Belvins et al obtained pulse oximetry measurements in mice anesthetized with varying concentrations of ketamine/xylazine or isoflurane under room air and 100% oxygen breathing conditions[5]. They demonstrated that anesthetized mice who did not receive supplemental oxygen became hypoxic, with an average SpO2 ranging from 70–80%. Still, despite being an invaluable telemetry tool, SpO2 measurements fail to accurately characterize the oxygenation of various tissues, instead only measuring the blood oxygen saturation supplying them.

Several techniques exist for non-invasive measurement of tissue oxygenation. Electron Paramagnetic Resonance (EPR) technique requires implantation of a lithium phthalocyanine (LiPc) probe into the tissue of interest, followed by measurements in a magnetic field. Liu et al and Abramovic et al obtained cerebral and skin EPR pO2 measurements, respectively, and demonstrated that type and concentration of anesthetic (isoflurane vs ketamine/xylazine) had a significant effect on tissue oxygenation[6],[7]. Still, the EPR technique has several important limitations; namely, implantation of LiPc probe likely alters tissue architecture, reports an average oxygenation value of the tissue that is in direct contact with the probe, and measuring a wider area requires implanting several EPR probes. The gold-standard method most utilized to measure tissue oxygenation is electrodes; however, these suffer from being invasive and slow responding.

Alternatively, optical techniques leveraging triplet state quenching by oxygen in luminescent probes can be employed. Both camera or fiber-optics have been used as the detector[8], [9]. These can reliably measure the full heterogeneity of pO2 distribution and/or the average values, *in vivo*. Using this technique, the quenching behavior of fluorescent dyes (excited singlet state) and phosphorescent dyes (excited triplet state) is described by the Stern-Volmer equation, such that the luminescent intensity is proportional to the luminescent decay time, based on the presence of oxygen[8, p. 1]. This technique, with the fluorescent probe PdG4 Oxyphor, specifically, has been used repeatedly to report extracellular oxygen levels in tissues, with an avalanche photo diode phosphorescence detector (APD, OxyLED, Oxygen Enterprises LLC Philadelphia, PA), calibrated to extract absolute pO2 readings from the lifetime-based quenching of PdG4[10]. Similarly, PpIX, another oxygen sensitive molecule based on triplet state quenching by oxygen, has been used to measure intracellular oxygenation levels[11], [12], [13]. Upon administration of aminolevulinic acid (ALA), protoporphyrin IX (PpIX) is metabolized in excess by the mitochondria in cells.

Here, we use the phosphorescence quenching of the oxyphor PdG4 to measure and contrast skin oxygenation in mice under three common isoflurane maintenance concentrations, as well as a commonly used dose of ketamine/xylazine. We further supplement our measurements with relative, intracellular tissue oxygenation via the delayed fluorescence from PpIX. The temporal profiles of skin tissue oxygenation demonstrate the importance of considering the choice of anesthetic in radiobiology studies and beyond.

## 2. Results

### 2.1 PdG4 Skin Oxygen Transient Measurements

**Figure 1:**
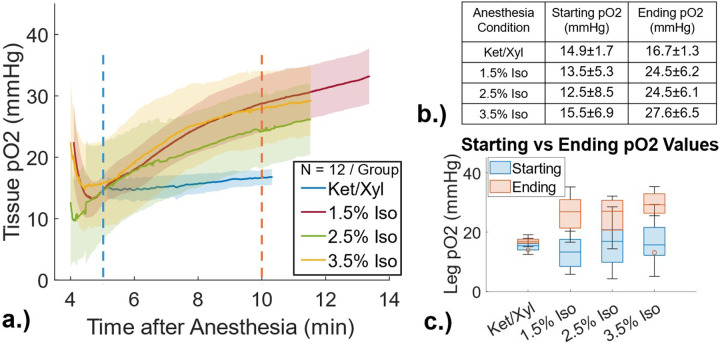
PdG4 measurements of skin oxygenation. a.) Average (N=12, 6 males and 6 females) temporal profile for each anesthesia condition. Shaded area is first standard deviation of data. b.) Average values at 5 minutes after induction and 10 minutes after induction. c.) Box plot comparison between two time points.

**Figure 2:**
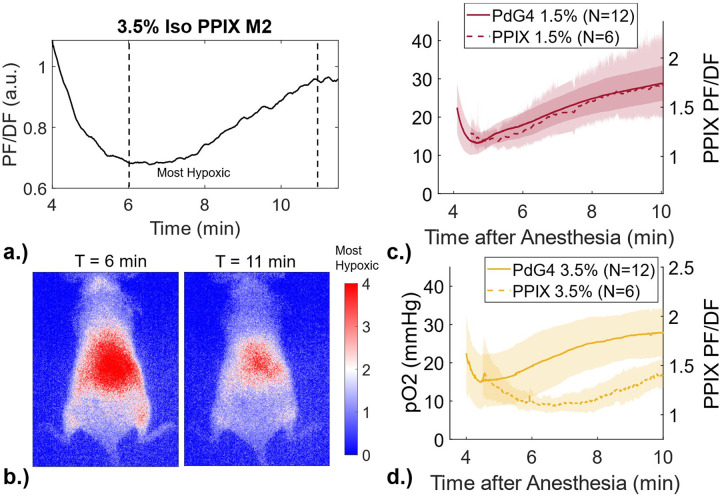
Qualitative measurements of skin oxygenation via PpIX. a.) Average temporal profile of leg skin ROI for mouse breathing 3.5% isoflurane and b.) full-field image of corresponding PpIX. c.) Average of 6 PpIX mice compared to 12 PdG4 mice breathing 1.5% Isoflurane. d.) Average of 6 PpIX mice compared to 12 PdG4 mice breathing 3.5% Isoflurane.

Figure 1 demonstrates the variability in skin tissue oxygenation as measured with PdG4 for the different anesthesia conditions under investigation and different timepoints measured. Figure 2a shows the average temporal profiles for mice breathing 1.5%, 2.5%, and 3.5% isoflurane with room air vs mice induced with ket/xyl. There is no significant difference between any of the four anesthesia conditions immediately after induction (5minutes), with an average pO2 of 14 ± 6 mmHg. Additionally, there was no significant difference between mice breathing different isoflurane percentages at 10minutes (average po2 = 26 ± 7mmHg), as summarized in Fig. 1b & c. However, ket/xyl was significantly different from all isoflurane breathing conditions at 10 minutes (mean pO2 16.7 ± 1.3 mmHg, p < 0.05). Tissue pO2 was significantly different at the start (5 minutes) vs end (10 minutes) for each anesthesia condition (p < 0.05). The 1.5% isoflurane breathing condition was the only case where male mice were significantly different from female mice, which is different than our previous findings[14].

### 2.2 Oxygen Transient Measurements via PpIX DF

An example of the whole-body tissue oxygenation during anesthesia, as measured temporally by the DF of PpIX, is shown in Fig. 2a. The 2D imaged maps of tissue oxygenation at the two relevant time points are shown in Fig. 2b, emphasizing the detail of information provided by this imaging method and where more hypoxia is observed at 6minutesimmediately after induction than at 11 minutes during the recovery. Temporal profiles of the averages of the 6 female, nude mice leg skin PF/DF are shown alongside the average temporal profiles measured with PdG4, in Fig. 2c and 2d, to demonstrate the deoxygenation and re-oxygenation of mice skin that happens with induction at 1.5% and 3.5% isoflurane concentrations, as seen by both measurement methods (intracellular and extracellular). Strong agreement between the PdG4 and PpIX data in the reoxygenation trends for mice breathing 1.5% isoflurane is demonstrated in Fig. 2b, with time to peak from the PdG4 measurements occurring at 4.7 ± 0.2 minutes vs 5.2 ± 0.4 minutes from the PpIX measurements. Time to peak hypoxia was found to be 4.7 ± 0.6 minutes for the measurements taken with the OxyLED/PdG4 for the mice breathing 3.5%, agreeing with the 1.5% measurements. The PpIX DF/PF results indicate peak hypoxia occurring at an average of 6.5 ± 1.2 minutes, as visualized by the data in Fig. 2b.

### 2.3 Histological Verification & Hyperspectral Imaging

**Figure 3:**
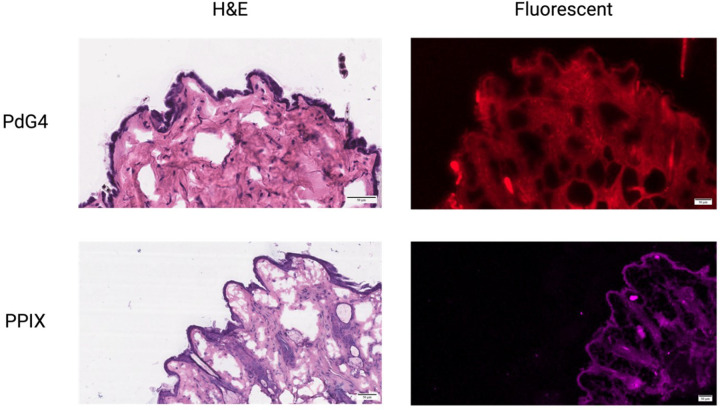
Comparison of H&E and fluorescent microscope localization of PdG4 and PPIXin skin layers.

The histological, fluorescent, images were used to verify the location of the PdG4 Oxyphor probe and ALA at the time of data acquisition/imaging. As expected, and shown in Fig. 3, the PdG4 was distributed extracellularly throughout the dermis and sub-cutaneous layers 1hr after injection but was notably absent from the epidermis. ALA, on the other hand, showed a more localized distribution within the epidermis as well as the cell-dense regions of the dermis. This is consistent with the expected intracellular distribution of ALA.

**Figure 4:**
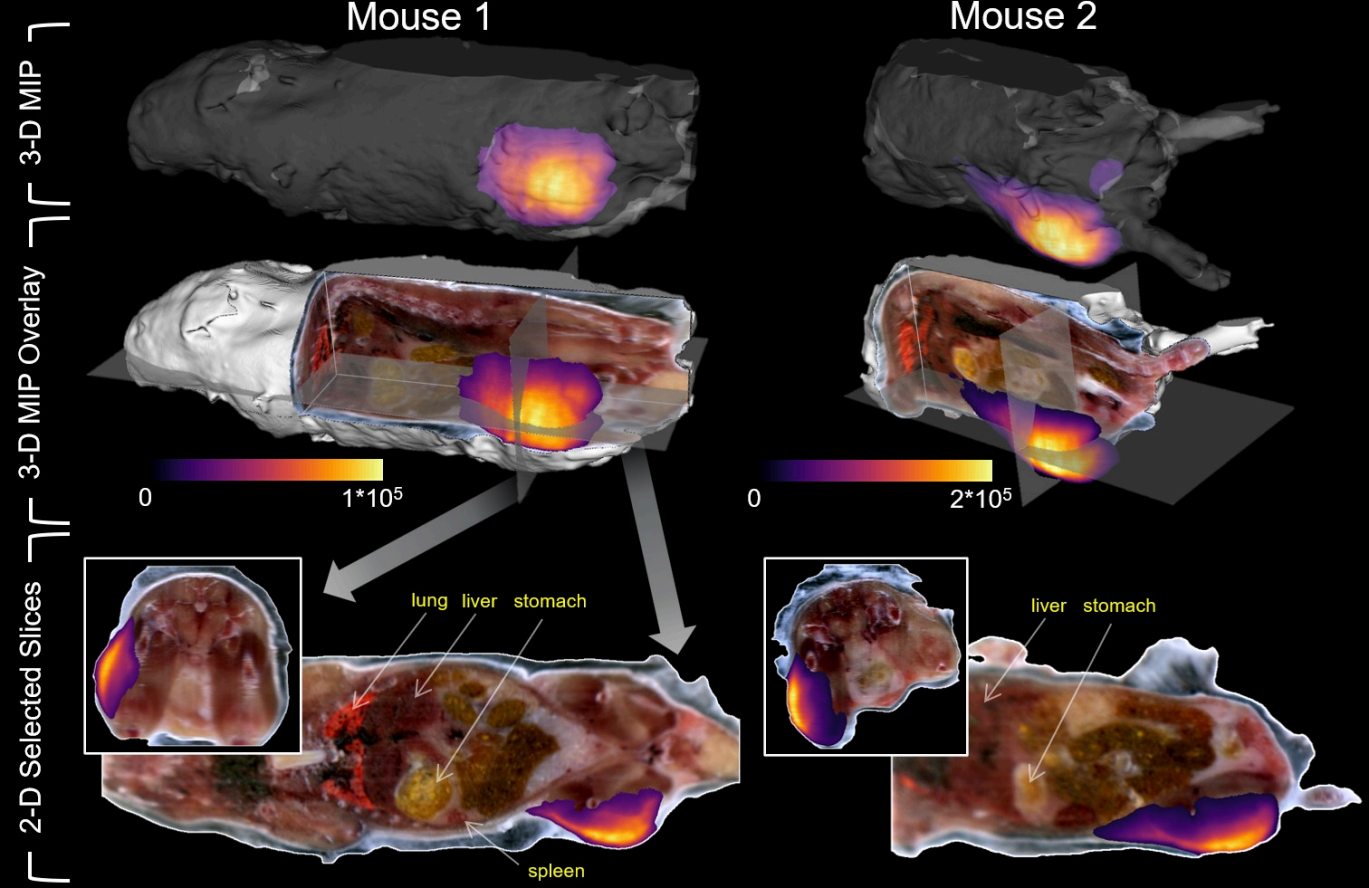
Whole-body fluorescence cryoimaging of two mice administered PdG4 in the hind limb. The top row shows maximum-intensity projection (MIP) images of the PdG4 fluorescence with a translucent rendering of the mouse surface. The second row shows the same MIP overlaid on the RGB volume of the mouse. Orthogonal 2-D slices (fluorescence overlaid on RGB) from the volumes are shown in the bottom row, as indicated by the gray orthogonal planes shown in the middle row.

Figure 4. Whole-body fluorescence cryoimaging of two mice administered PdG4 in the hind limb. The top row shows maximum-intensity projection (MIP) images of the PdG4 fluorescence with a translucent rendering of the mouse surface. The second row shows the same MIP overlaid on the RGB volume of the mouse. Orthogonal 2-D slices (fluorescence overlaid on RGB) from the volumes are shown in the bottom row, as indicated by the gray orthogonal planes shown in the middle row.

Figure 4 demonstrates the distribution of PdG4 throughout the entire body of the mouse 1 hour after injection using a novel imaging method. Again, as expected, the PdG4 can be visualized throughout the outer leg. This is, to our knowledge, the first characterization of PdG4 with three different imaging methods.

## 3. Discussion

It is well established that tissue radiosensitivity is dependent on the relative oxygenation of that tissue, with the highest dependance below a PO_2_ of 20 mmHg. Here we demonstrate that type and time under anesthesia are critical modulators of skin oxygenation in mice. We show that ket/xyl anesthesia results in a more moderately hypoxic, but temporally stable, level of tissue oxygenation, whereas isoflurane anesthesia is associated with more variable tissue oxygenation levels.

Looking at the temporal characteristics of the PdG4 measurements, we see agreement in time-to-peak between mice breathing 1.5% and 3.5%. Comparing the relative changes in tissue oxygenation measured via PdG4 vs PPIX, we see a slightly delayed time to peak tissue hypoxia using PPIX measurements. Interestingly, this delay appears to prolong with increasing anesthetic concentration. We propose that this is likely owing to PdG4 and PPIX representing the oxygenation of extracellular/interstitial and intracellular compartments, respectively, and offer two hypotheses for a potential mechanism. First, given that the oxygen concentration of the intracellular compartment is a function of free diffusion with the extracellular space as well as oxygen consumption by the cell, a delayed hypoxia of the intracellular compartment can be caused by changes in either factor. Isoflurane has been shown to increase membrane bilayer permeability, while at the same time slowing intracellular respiration [15]. Reduced intracellular oxygen use can at least partially explain the delayed time to peak hypoxia.

Though few studies exist that have evaluated skin oxygenation under varying anesthetic conditions, this, to our knowledge, is the first report of an optical technique used to create temporal skin oxygenation profiles under varying anesthesia conditions in mice. Optical techniques have several advantages over previously established methods, such as EPR or electrode sensors. First, the optical reporters cause minimal tissue disruption. In instances where the reporter is injected into the target tissue locally the required injection volume is small which together with the highly water-soluble nature of the PdG4 make damage to tissue structure less likely. Importantly, PdG4 can be administered intravenously (IV), without toxicity, thus allowing for tissue oxygen measurements without manipulating the target tissue. In the black mice (C57BL/6), significant pigmentation of the skin makes for a suboptimal signal-to-noise ratio for oxygen measurements after IV administration of PdG4, thus necessitating local injection. However, we have successfully and repeatedly measured oxygenation in other organs of black mice after IV PdG4 administration, and Esipova et al demonstrate similar success with measuring skin tumor oxygenation in non-black mice[16].

The second advantage of this technique is demonstrated by the finding that when isoflurane anesthesia is used, tissue oxygenation is more variable both between animals and within the same animal with time. For radiation damage experiments, this points to an emerging need to control – or at least standardize – practices that will affect tissue oxygenation at the time of irradiation. The optical technique allows us to measure this, owing to the fast speed of the reporter and requiring a simple laser-detector pair for oxygen measurement. Where real-time measurement is not possible, the data presented here can serve as a general guide to estimating skin oxygenation in black mice and under the prescribed anesthetic conditions. For instance, to avoid hypoxia when using isoflurane anesthesia, we show that waiting at least 10minutesafter commencing anesthesia can bring the skin pO2 well above the 20mmHg threshold. At the very least, controlling the time from anesthesia start to irradiation is indicated.

One potential limitation of the PdG4 reporter, similar to EPR, is that the oxygen measurements are limited to the extracellular compartments since PdG4 cannot cross the cell membrane. Though extracellular tissue oxygenation is a valuable indicator of overall oxygen status, an interesting question is whether this correlates with intracellular oxygen dynamics. Here, we were able to supplement extracellular PdG4 measurements to intracellular PpIX measurements. We show that PdG4 and PPIX temporal oxygen profiles for 1.5% and 3.5% isoflurane anesthesia correlate closely.

There are several important limitations to our findings that warrant discussion. First, given that response to anesthesia is driven by a host of physiologic parameters, caution should be taken when extrapolating the oxygenation values and temporal dynamics presented here to different species of mice or larger animals. Still, our results demonstrate the importance of taking tissue oxygenation into account when designing, conducting, and interpreting radiobiological studies, especially when investigating radiation damage. Though the present study was focused on mice, obtaining temporal tissue oxygenation profiles like those presented here is also of particular interest for larger animals, where multiple modes of anesthesia (injectable and volatile) are typically used for induction and maintenance of anesthesia.

A second limitation of PdG4 oxygen measurements is relatively poor spatial specificity. As demonstrated by our fluorescent and hyperspectral images, the highly water soluble PdG4 molecule readily diffuses across the tissue plane it is injected in. As a result, when an optic fiber pair is used to measure tissue oxygenation, the actual oxygenation value is an average of the width and depth of the entire area that is excited by the laser and detected by the sensor. For instance, though our mice received subcutaneous injections of PdG4, it is likely that the tissue oxygenation values are an average of dermal, sub-cutaneous, and muscular interstitial oxygen levels. This limitation can be partially overcome by utilizing a camera to image the PdG4 decay rather than a fiber optic detector which would provide helpful spatial information about the area being measured as previously described[17].

Finally, PpIX PF/DF images are useful in providing intracellular oxygenation level temporally across the mouse skin surface. It should be noted that skin oxygenation varies depending on the region of interest, another consideration that should be factored in when planning radiation therapy studies. Further investigation into common irradiation sites should be completed for a better understanding of this effect on mouse oxygenation status. The major limitation of PpIX DF/PF measurements lie in that they only provide a relative oxygen status of the tissue. Previous studies have tried to correlate intracellular measurements to absolute values using other fluorescence probes or electrodes; however, the fundamental problem of the probes being in different places remains. Additionally, these probes are similar to the PdG4 measurements in that they are distributed throughout the tissue when injected IP, such that the images reflect a sampling of the excited tissue, lying in the penetration range of the excitation laser.

The findings presented here also shed light on yet another factor that is likely a source of variability in ultra-high dose rate and FLASH effect studies, an active area of research in RT. Multiple reports have recently shown that use of supplemental oxygen as anesthesia carrier gas negates the FLASH effect, owing to increased tissue oxygenation[14]. Radiolytic oxygen consumption has been proposed as a mechanism to explain the FLASH effect with many theoretical and experimental studies reported in recent years. A search of oxygen and FLASH on PubMed returns 212 such publications with authors supporting and refuting the effects of oxygen and in-vivo measurements under various anesthesia conditions. This is the first quantitative report of tissue oxygenation as a function of anesthesia type and time after induction of anesthesia, with an oxygen measurement method that can be used during irradiation. As noted above, differences in tissue oxygen levels for a single anesthetic regiment may vary be a factor of 3 or more as a function of time alone. Oxygen consumption during irradiation is a precursor to radical formation and subsequent cellular damage and has been reported to be a function of the initial pO2, both in-vitro and in-vivo. Since the initial, pre-irradiation p02 is a function of anesthesia type and time after induction, these parameters need to be carefully considered and controlled for in studies seeking to exploit the FLASH normal tissue sparing effect during UHDR irradiation.

## 4. Methods

### 4.1 PdG4 Oxyphor Measurement of Tissue Extracellular pO_2_

Twelve C57BL/6 (black) mice (6 males, 6 females) were used for measurement of subdermal oxygen levels for each of the anesthetic conditions (N = 48). Previous studies demonstrated that male and female mice breathing room air showed no significant difference in leg pO2 measurements [14], [18]; therefore, all mice were anesthetized with room air. The mice were shaved and 0.05mL of 20uM PdG4 dissolved in phosphate-buffered saline (PBS) (Corning, Corning, NY) was injected subcutaneously into the leg of the mice one hour prior to measurements.

For the mice breathing isoflurane through a nose cone, each mouse was induced for three minutes at 3% isoflurane delivered at 500 ml/min. After three minutes, the isoflurane concentration was switched to either 1.5%, 2.5%, or 3% at 100 ml/minutes and maintained at that concentration and flow rate for the remainder of the session, as shown in Fig. 1. For the ketamine/xylazine cases, the mice were weighed and injected with 100/10 mg/kg ket/xyl and measurements began after they were induced (typically within 5 min).The phosphorescence time decay of PdG4 was measured with a the OxyLED system (Oxygen Enterprise, Philadelphia, PA), which has been used for *in vitro* and *in vivo* oxygen measurements [19], [20], [21]. The system sampled a 1cm diameter area of the mouse leg at a rate of 2 Hz. The OxyLED system comprises a fiber pair that serves dual functions: it acts as a source for fluorescence excitation using a pulsed, red light (637 nm) and as a collecting fiber connected to an avalanche photodiode detector (ADP). This setup was positioned approximately 5mm from the skin surface, and the oxygen pressure (in mmHg) was continuously recorded for at least ten minutes. Core body temperature was maintained at 37.5°C via a heating pad.

All mice were obtained from Jackson Laboratories, and animal experiments were approved by Dartmouth College Institutional Animal Care and Use Committee under Protocol 00002257(m6a) and all methods were performed in accordance with the relevant institutional guidelines and broader regulations. At time of euthanasia mice were deeply anesthetized using 5% isoflurane and euthanized by cervical dislocation and subsequent creation of pneumothorax. All methods adhere to ARRIVE guidelines.

### 4.2 PpIX Measurement of Tissue Cellular pO_2_

To compare to and validate the extracellular subdermal oxygenation measured with the OxyLED, the delayed fluorescence (DF) of PpIX, previously used to measure relative skin tissue oxygenation in mice[22], was imaged. Endogenously produced PpIX DF is known as an intracellular oxygen sensor that can be used *in vivo*, and the oxygen reporting capability using DF from PpIX has been reported in many studies [12], [13], [23].

**Figure 5:**
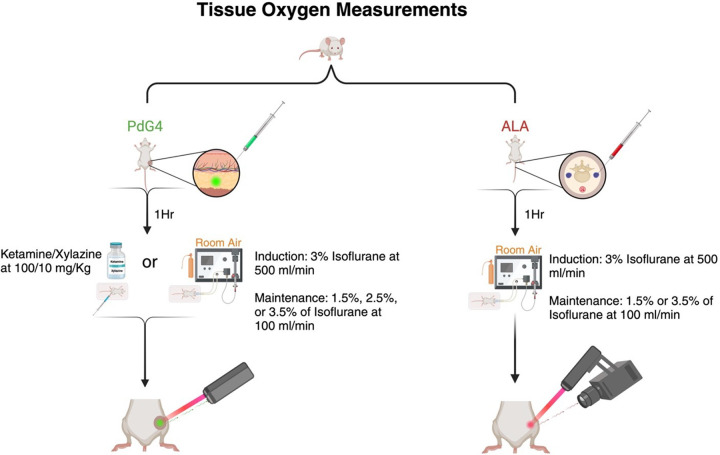
Study design. The two columns show the injection of probes and the anesthesia and gas conditions under which measurements were taken for the two oxygen measurements.

6 NU/J (athymic nude) female mice (Charles River Labs, Wilmington, MA) were used in this study. On the day of imaging, ALA (Sigma-Aldrich, MO) dissolved in PBS was injected intraperitoneally (250 mg/kg) two hours prior to imaging. At the time of imaging, mice were anesthetized using the same procedure described for the PdG4 measurements in Section 2.1 and shown in Fig. 5, and images for the cases of mice breathing 1.5% oxygen and 3.5% isoflurane were captured with an intensified CMOS camera, as described by Petusseau et al[22]. A sequential acquisition of prompt fluorescence (PF) and DF of PpIX allowed for an effective frame rate of 10 fps, facilitating real-time reconstruction of a normalized hypoxia image (i.e., DF/PF). This notation is utilized to normalize the DF signal, taking into consideration the tissue’s optical properties and PpIX concentration. DF/PF was inverted to correlate data with PdG4 measurements for ease of understanding throughout this paper.

### 4.3 Data Processing

PdG4 Oxyphor is a well-characterized oxygen probe that allows quantification of molecular oxygen across the biological range of pO2[16]. Its phosphorescent lifetime varies with oxygen concentration, making it a robust and translatable probe for oximetry. The OxyLED is calibrated to readout quantitative oxygen levels in mmHg, so the temporal profiles of skin oxygenation for each mouse were direct outputs of the system and plotted in time. All six male and six female mice data were averaged for evaluation. Tissue oxygenation levels were compared immediately after the breathing conditions of the mice were altered to those observed after a period of equilibration. This comparison was made by using averages and first standard deviation of at least 50 data points centered at 5 minutes and 10 minutes after induction.

PpIX ratiometric fluorescence signal time profiles were created from the average of a 30-pixel diameter region of interest (ROI) corresponding to the approximate sampling area of the OxyLED on the leg of each mouse, though whole-body skin tissue oxygenation can be visualized with this methodology. The PpIX temporal profiles from the six imaged mice were averaged and compared to the PdG4 qualitatively.

### 4.4 Histological & Whole-Body Fluorescence Cryoimaging Verification of Localization

To verify distribution of PdG4 and PPIX within the skin tissues, a C57BL/6 mouse and a NU/J mouse received subcutaneous and intraperitoneal injection of PdG4 and PPIX, respectively. Another two mice served as controls. Mice were sacrificed 1 hour after injection, skin biopsies were obtained from the leg and frozen in optimal cutting temperature compound (OCT) (Fisher Scientific, Pittsburgh, PA). Eight serial frozen sections were obtained from each mouse, four of which were stained with H&E and four which underwent fluorescent microscopy with a Zeiss LSM880 Airyscan (Zeiss, Oberkochen, Germany).

We used whole-body hyperspectral fluorescence cryo-imaging to visualize the distribution of PdG4 in animal models. This technique autonomously sections and images whole frozen specimens to recovery the 3-D distribution of fluorescent reporters overlaid on specimen anatomy, as detailed previously[24]. In this study, C57BL/6 mice were administered 0.05 mL at 20 μM of PdG4 via subcutaneous injection in the left hind leg. One hour after administration, the animal was euthanized, embedded in OCT compound and frozen to −20°C before being mounted in the cryo-imaging instrument. Specimens were sectioned in 100 μm thick sections. After each section was removed, the specimen was illuminated sequentially with a white light source to acquire anatomical RGB images and a 635 nm laser (Intense Co, New Brunswick, NJ) to excite PdG4 and image its fluorescence emission. For the latter, light remitted from the specimen passed through a 750 nm dichroic mirror and a 780 nm long pass filter before being recorded with a sCMOS camera (Edge 4.2, PCO, Bavaria, Germany). The RGB and PdG4 fluorescence image stacks were processed and rendered into 3-D volumes using 3D Slicer (http://www.slicer.org).

### 4.5 Statistical Analyses

The tissue oxygen measurement data was analyzed using independent sample t-tests. All analyses were conducted using Matlab.

## 5. Conclusion

Anesthetic type has long known to play a major role in tissue oxygenation; however, this is the first report, to our knowledge, characterizing the temporal dynamics, using multiple imaging modalities and biological reporters, of tissue oxygenation due to changes in anesthetic condition. The temporal dynamics in tissue pO2 that occur from deep anesthesia with isoflurane are significant, with a low value at early times (4–6 min) near 14–16 mmHg and rising to a higher value nearer 25–27 mmHg at 10 minutes in mice. This can be contrasted with a steady low pO2 value when mice are induced by ketamine/xylazine nearer 12 mmHg. There may be an interesting difference in cellular versus extracellular pO2 at the deepest isoflurane anesthesia at 3.5%, however this likely requires further study. Perhaps most importantly is that there is range of values with individual mice having high variation, and the deepest anesthesia uses of isoflurane can result in the skin pO2 dropping below the threshold for radiobiological hypoxia (pO2 < 10 mmHg) in some animals, suggesting that this use must be done with caution in radiation research studies. The later times of gas anesthesia appear to have a more consistently high normal pO2 value of 24–26 mmHg in the skin.

## Data Availability

The datasets generated during and/or analysed during the current study are available from the corresponding author on reasonable request.
